# Acknowledgement to Reviewers of *Sensors* in 2013

**DOI:** 10.3390/s140203797

**Published:** 2014-02-25

**Affiliations:** *Sensors* Editorial Office, MDPI AG, Klybeckstrasse 64, CH-4057 Basel, Switzerland

The editors of *Sensors* would like to express their sincere gratitude to the following reviewers for assessing manuscripts in 2013:

Abang, Ada

Abawajy, J. H.

Abbas, Hazem M.

Abbaszadeh, S.

Abdelgawad, Amr Atef

Abdelkefi, Abdessattar

Abdulhalim, Ibrahim

Abe, T.

Abe, Takashi

Abe, Tomoki Abe

Abeywardena, Dinuka

Abramchuk, George

Abramovich, Haim

Acar, Evrim

Acosta-Jiménez, Antonio J.

Acquaviva, Andrea

Adam, Vojtech

Adamchuk, Viacheslav I.

Adamiak, Anna

Adams, Martin

Adams, Teresa M.

Adda, Mo

Adekunle, Andrew A.

Adewumi, Aderemi

Adrien, Briod

Aeron, Shuchin

Afzal, Adeel

Agafonov, Vadim

Agapiou, Athos

Aggarwal, Amit

Agili, Sedig S.

Aglyamov, Salavat R.

De Agostino, Mattia

Agüera Vega, Juan

Aguiar, Javier M.

Aguilar, Juan José

Ahmadi, Ali

Ahmed, Minhaz Uddin

Ai, Shiyun

Aidala, Katherine

Aita, André Luiz

Akhavan, Hooman

Akitsu, Tetsuya

Aksyuk, Vladimir

Al-Mashat, Laith

Al-Sarawi, Said F.

Alba-Flores, Rocio

Albert, Amos

Albert, Mark V.

Alberto, Nelia

Alcock, Paul

Alexandridis, Alex

Alfaro, Sadek C.A.

Alfredson, Henrik

Ali-Löytty, Simo

Ali, Syed Taha

Alici, Gursel

Allan, Chan C.K.

Allende, Ana

almayali, yahya

Alonso-Fernandez, Fernando

Alonso, Eduardo

Alonso, Jesús B.

Altun, Kerem

Alvarez, Juan Carlos

Amditis, Angelos

Amezcua-Correa, Rodrigo

Amft, Oliver

Amigo, José Manuel

Amin, Ashraful

Amir, Mohammad

Amiruzzaman, Md

Amitat, Yassine

Amouri, Abdulazim

An, Fengwei

Ananth, Z.

Anderson, D. M.

Anderson, Sharolyn

Ando, Bruno

Andre, Lourenco

André, Paulo

Andreescu, Silvana

Andreoni, Giuseppe

Andrieux, Fabrice

Andújar, José M.

Anfossi, Laura

Angelidis, Pantelis A.

Anguita, Davide

Angulo, Itziar

Angus, Doug

Annamdas, Venu Gopal Madhav

Antfolk, Christian

Anthony, Carl J.

Antonopoulos, Angelos

Antonovskaya, Galina

Antunes, Paulo

Anvar, Amir Mohammad

Aoyagi, Takahiro

Aparicio, S.

Apetrei, Constantin

Aphale, Sumeet S.

Apps, Peter

Arai, Nobuaki

Arakawa, Takahiro

Arandjelovic, Ognjen

Araujo, Helder

Arayici, Yusuf

Arce, Gonzalo R.

Arcelus, Amaya

Ardid, Miguel

Arduini, Fabiana

Arena, Antonella

Arena, Mario Eduardo

Ariga, Katsuhiko

Armenta, S.

Arnó, Jaume

Arnold, Steve

Aroca, Rafael

Arregui, Francisco J.

Arribas, Javier

Arruda, Valeria

Artal, Pablo

Arya, Sunil

Arya, Sunil K.

Asato, Yuji

Asensi, Marise D.

Askraba, Sreten

Aslanides, Ioannis M.

Assunção , Pedro

Astráin Escola, José Javier

Atkinson, Rob

Atsushi, Mitani

Attoh-Okine, Nii O.

Atulasimha, Jayasimha

Atzori, Luigi

Auat Cheein, A.

Auat-Cheein, Fernando

Avolio, Alberto P

Axinte, Eugen

Azariadis, Philip N.

Azencott, Chloé-Agathe

Azzari, George

Azzopardi, George

Baba, Kensuke

Bäck, Jaana

Bae, Joonbum

Bagan, Hasi

Bahillo, Alfonso

Bai, Jinhe

Bai, Shengqiang

Baiardi, Fabrizio

Bakonyi, Imre

Bakr, Bilal Abu

Baktir, Selcuk

Balandin, Alexander A.

Balas, Costas

Balas, Valentina E.

Balibar, Sébastien

Ballester, Miguel ángel Ferrer

Balogh, Nandor

Bang, Ole

Banitsas, Konstantinos

Bankova, Vassya

Bansmann, Joachim

Barbieri, Pierluigi

Barbosa, Rui M.

Barden, John M.

Barralon, Pierre

Barranco, Francisco

Barreira, Eva

Barriera, Tiago

Barros, Gracian Trivino

Barroso, Margarida

Barsocchi, Paolo

Bartelt, Hartmut

Bartlett, Rebecca

Bartolini, Sandro

Barton, Richard J.

Bartsch, Heike

Bartussek, Jan

Barut, Miloš

Barzaghi, Riccardo

Bashkatov, Alexey Nikolaevich

Basso, Bruno

Bauer, Franqois

Bauer, Martin

Bauriegel, Elke

Bausells, Joan

Bayarri, Miguel Souto

Beauchamp, Jonathan

Becher, Christoph

Begout, Marie Laure

Begovich, O.

Behdad, Nader

Beheim, Glenn M.

Beheshti, Soosan

Belabbas, Boubeker

Belbachir, Ahmed Nabil

Bell, David

Bellavista, Xiao-Quan

Belsey, Natalie

Bende-Michl, Ulrike

Benecke, Wolfgang

Benedetti, Matteo

Benet, Gines

Benezeth, Yannick

Benzo, Maurizio

Berberich, Jason

Berckmans, D.

Beresna, Martynas

Beretta, Giovanni

Berger-Vachon, Christian

Bergmann, Jeroen H. M.

Bernards, Matt

Bernini, R.

Berretti, Stefano

Bersani, Ferdinando

Berselli, Giovanni

Berthiau, Gérard

Bestak, Robert

Bevilacqua, Alessandro

Bevly, David M.

Beyerer, Jürgen

Bhadra, Jolly

Bhattacharyya, Abhijit

Bickel, Bernd

Bidart, Christian

Biener, Gabriel

Binions, Russell

Birk, Wolfgang

Bisceglie, Maurizio Di

Björninen, Toni

Blackman, Chris

Blanc, Philippe

Blanco, Sonia García

Blasco, Josiva

Bläuer, Christine

Boehnen, Chris B.

Bolitho, Megan

Bolognesi, Andrea

Bondavalli, Paolo

Bondia, Jorge

Bonelli, Pablo R.

Bonev, Ilian

De Boor, J.

Borges, Paulo V. K.

Borini, Andrea

Borisov, S.M.

Borjas, Santos Demetrio

Miranda

Bortnowska, Grażyna

Bosselmann, Thomas

Bostater, Charles R.

Botella, Guillermo

Boudaoud, Sofiane

Boudon, Frédéric

Boukabache, Hamza

Boukallel, Mehdi

Boukas, El-kébir

Bouma, Henri

Bouwmans, Thierry

Bovtun, Viktor

Boyle, David

Bradley, Colin

Braem, Bart

Bragos, Ramon

Brandl, M.

Braun, Daniel

Brauns, E.

Breda, Marco

Bregestovski, Piotr

Breier, Matthias

Bresler, Yoram

Breton, Marc D

Brimblecombe, Peter

Brittain, William J.

Brocca, Luca

Broche, Lionel

Brocker, David

Brodaric, Boyan

Brosseau, Christa

Brown, Danielle

Bruce, Neil D. B.

Bruder, Gerd

Brukman, Matthew

Brunner, Andreas J.

Brunner, Clemens

Brutti, Alessio

Bu, Jiajun

Bucksch, Alexander

Buddenbaum, Henning

Bui, Nicola Bui

Buonasera, Katia

Burger, Arnold

Burleson, Wayne P.

Büttgenbach, Stephanus

Cabestany, Joan

Cabon, Beatrice

Cahill, Brian P.

Cai, Yinghao

Caiti, Andrea

Caldarelli, Stefano

Caldelli, Roberto

Caleffi, Marcello

Caliano, Giosue

Callahan, Brian P.

Calleja, M.

Calvin, Lau

Camci-Unal, Gulden

Campbell, Zachary T.

Campopiano, Stefania

Camuffo, Dario

Canale, A.

Cañas Plaza, José María

Canosa, Roxanne L.

Cantarero Sáez, Andrés

Cantó, Enrique

Cantoni, Lorenzo

Cao-Paz, Ana M.

Cao, Anyuan

Cao, Feilong

Cao, Haishi

Cao, Huasong

Cao, Yu

Capella Hernández, Juan Vicente

Capilla, Rafael

Capitani, Donatella

Cardimona, D.A.

Cardinale, Massimiliano

Caria, Maria

Carlen, E. T.

Carlen, Edwin T.

Carlomagno, Giovanni Maria

Carmo, João Paulo Pereira Do

Carmona-Martínez, Alessandro A.

Carmona, Jean-Cluade

De Carolis, Berardina

Carotenuto, Riccardo

Carpino, G.

Carrasco, Miguel

Carty, Christopher P.

Carvalho, Sabrina

Casale, Pierluigi

Casanova, Jose Luis

Casciati, Fabio

Caseli, Luciano

Casini, Marco

Casson, Alexander J.

Casteigts, Arnaud

Castrillon, Modesto

Catalfamo, Paola

Cavalcanti, George Darmiton C.

Cavaleiro, Ana M.V.

Caviglione, Luca

Cayuela, J.A.

Cecchi, Francesca

Cela, Sara Abalde

Cennamo, Nunzio

Cense, Barry

Ceres, Ramon

Ceriotti, Matteo

Cerqueira, Eduardo

Cesarino, Ivana

Cha, Suk Won

Chachalis, Demosthenis

Chae, Youngcheol

chalhoub, gerard

Chamanzar, Maysamreza

Chan Jun, Seong

Chan, Chia-Tai

Chan, Edwin P.

Chan, Hau Ping

Chan, Weng

Chandler, Josephine R.

Chandran, Vinod

Chanerley, Andrew A.

Chang, Carl.W.

Chang, Chien-yi

Chang, Chih-Yuan

Chang, Chih-Yung

Chang, Edward Yi

Chang, Jenq -Yang

Chang, Kang-ming

Chang, Kuan-Tsung

Chang, Shao-Hsia

Chang, Woo-jin

Chang, Rwei-Ching

Chaouki, Jamal

Chapko, Alexandra

Chapman, C. Elaine

Chattopadhyay, Arunabh

Chatzandroulis, S.

Chau, Lai-Kwan

Chau, Yuen

Chaves-González, Jose M.

Checchin, Paul

Chehimi, Mohamed

Chembo, Yanne K.

Chen, Chao Yu Peter

Chen, Chen-yuan

Chen, Chung-Hao

Chen, Cynthia

Chen, Duan-yu

Chen, Fang-Chung

Chen, Fangjiong

Chen, Felix

Chen, Guang

Chen, Jiajun

Chen, Lujie

Chen, Min

Chen, Nan-kuei

Chen, Peng

Chen, Qiang

Chen, Ray T.

Chen, Rong-Sheng

Chen, S. Y.

Chen, Shu-qing

Chen, Shunfeng

Chen, Wei

Chen, Wei-Fan

Chen, Xiangdong

Chen, Xiangfei

Chen, Xianping

Chen, Xuefeng

Chen, Xuemin

Chen, Yenlin

Chen, Yuwei

Cheneler, David

Cheng, H

Cheng, Jen-Yuan

Cheng, Jiangtao

Cheng, Ming-Cheng Mark

Cheng, Shi

Cheng, Shunfeng

Cheng, Teng

Cheng, Wei-chen

Cheng, Wood-hi

Cheng, Xuanhong

Cheng, Yang

Chesmore, David

Chessa, Stefano

Chestek, Cindy

Chiabrando, F.

Chiang, Jen-Shiun

Chiang, John Y.

Chiang, Kai-Wei

Chikamune, Wada

Chin, B.A.

Chin, Craig A.

Ching, Tak-shing

Chinzei, Kiyoyuki

Chipara, Octav

Chiribiri, Amedeo

Chiu, Alan W.L.

Chiu, Tai-chia

Chng, Shu Sin

Cho, Beongki

Cho, Gihwan

Cho, Ho-Shin

Cho, Jae Yoong

Cho, Josh

Cho, Nam-joon

Cho, Soojin

Choi, Jae-Won

Choi, Jun Kyun

Choi, Manyong

Choi, Seibum B

Chondrou, Charline

Choo, Jaebum

Chou, Li-Der

Choy, Sue Lynn

Christin, Delphine

Christophersen, M.

Chrostowski, Lukas

Chu, Shu-Chuan

Chung, Ching-che

Chung, Hoam

Chung, Sun-ok

Chung, Youngjoo

Ciaccheri, Leonardo

Ciampa, Francesco

Cikajlo, Imre

Cimalla, Volker

Civet, Yoan

Claesen, Luc

Clarkson, Sean

Cleland, Ian

Coblentz, Wayne K.

Cocconcelli, Marco

Cochrane, Cédric

Cohen, Kelly

Cole, Melanie

Colitti, Walter

Collin, Jussi

Collins, Stephen

Comyn, Timothy

Conforto, Silvia

Constable, Steven

Conti, Vincenzo

Coo, Lilibeth

Corcoran, Peter

Cordero Fuertes, Juan Antonio

Cordes, Kai

Corigliano, Alberto

Corless, Robert M.

Corrado, Costa

Correa, Christian

Correia, José Higino Gomes

Corsi, Cristina

Cortes, Pedro

Cosh, Michael

Cosnier, Serge

Costa Branco, P.j.

Costa García, Agustín

Costa-García, Agustín

Costas, Vassilakis

Costin, Andrei

Cotos, José M.

Courtney, Charles

Coutier, Caroline

Covington, James

Coyle, Shirley

Cozzolino, Daniel

Cragg, Peter J.

Craig, Cathy

Crandall, Aaron

Crane, Nathan B.

Craven, Michael P.

Creţ, Octavian

Cristani, Marco

Cristescu, Rodica

Crouse, David F.

Cuadras, Angel

Cui, Daxiang

Cui, Lina

Cui, Shumao

Cui, Yong

Culbertson, Christopher T.

Culshaw, Brian

Curiel-Esparza, Jorge

Cusano, Andrea

Cywinski, Piotr

Czajkowski, Jakub

D'Ippolito, Filippo

D'alessandro, Brian

Dahnoun, Naim

Dai, Gaoliang

Dai, Ji Yan

Dai, Kaoshan

Dale, Laura M.

Damaren, Christopher J.

Dan, Yaping

Danger, J.-L

Daniele, Passeri

Danieli, Ernesto

Daribo, Ismael

Darie, Costel

Dauksevicius, Rolanas

Dawson, Tom

Day, Iain J.

Day, Stephanie S.

De Baere, Ives

de Bruin, Eling Douwe

De Cesare, Fabrizio

De Gennaro, Gianluigi

De La Cruz, Carlos A.

de la Escalera, Arturo

De la Oliva, Antonio

De La Puente, Paloma

De Los Santos Valladares, Luis

De Maeztu, Leonardo

de Paul Obade, Vincent

De Pedr, Teresa

De Poorter, Eli

De Souza, Paulo

De Vries, W.h.k.

Deambrogio, Lina

Debar, Hervé

Debener, Stefan

Debono, Carl James

Décima, P.

Deckert, Alice

Dedousis, Athanasios P. Dedousis

Deenik, Jonathan

Deflandre, Bruno

Defoirdt, Tom

Del Cerro, Jaime

Del Valle, Manuel

Dell'olio, Francesco

Delmotte, François

Delnavaz, Aidin

Deng, Le

Deng, Mingxi

Deng, Weihong

Denis, Proshlyakov

Dennis, John Ojur

Derossi, A.

Descrovi, Emiliano

Desertot, Mikael

Deshi, Li

Desouza, Guikherme

Dessein, Arnaud

Dessers, Ezra

Devillers, Laurence

Devy, Michel

Dhadwal, Harbans S.

Di Natale, Corrado

Di Pasquale, Fabrizio

Di Pietrantonio, Fabio

Dias, José Mendonça

Dias, Teresa Galvão

Díaz, Xavier Armengol

Dickenson, Robert

Dickert, Franz

Dilli, Zeynep

Dimitra, Vasileios K.

Dimitri, Breda

Dincalp, Haluk

Ding, Bin

Ding, Hui

Ding, Jow-Lian

Ding, Kang

Ding, Shijia

Dios, J.R. Martinez-de

Dipper, Nigel

Divos, Ferenc

Doblin, Martina

Dobosz, Marek

Dobre, Ciprian

Dode, Margot Alves Nunes

Dogaru, Radu

Dogramadzi, Sanja

Doherty, Sean

Doll, Joey

Dominguez Brito, Antonio Carlos

Domínguez Renedo, Olga

Domínguez, Juan Jesús García

Dommermuth, Franz

Dong-Hwan, Hwang

Dong, Daming

Dong, Hua

Dong, Liang

Dong, Lili

Dong, Wen

Dong, Xinyong

Dong, Yabo

Dong, Yanchao

De Donno, Danilo

Donzella, Valentina

Dorogin, Leonid M.

Dorrío, Benito V.

Doukas, Charalampos

Douligeris, Christos

Dourado, António

Douša, Jan

Chen, Shen-En Dragon, Mauro

du Burck, Frederic

Du, Ke

Du, Ping

Du, Yanan

Du, Yanfeng

Du, Yu

Duan, Huang Feng

Duan, Jicheng

Duarte Ortigueira, Manuel

Dubey, Venky

Duchateau, Nicolas

Duffy, Carol

Dufour, Suzie

Duggan, Ian C.

Dunbar, Alan

Duque, Nestor D.

Durduran, Turgut

Dutta, Debaditya

Dutta, Prabal K.

Dutta, Ritaban

Duviella, Eric

Duy, Truong Vinh Truong

Dyo, Vladimir

Dzyuba, Sergei

Eaton, Mark Jonathan

Edwards, Jonathan

Ehlert, Detlef

Ehmann, Kornel

Ehsan, S.

Ehtezazi, Touraj

Eichholz, Jörg

Eielsen, Arnfinn

Eizenman, Moshe

Ekström, Mikael

El Saddik, Abdulmotaleb

El-Gohary, Mahmoud

El-Kadri, Oussama

El-Osery, Aly I.

El-Taher, Atalla

El-Tawab, Samy

Eliades, Demetrios

Elkins, Aaron C.

Elliott, Hannah

Elyan, Eyad

Emelchenko, G.A.

Emmanouilidis, Christos

Van Den Ende, D. A.

Endo, Tatsuro

Engesser, M.

Englebienne, Gwenn

Enikov, E. T.

Enright, John

Epaarachchi, Jayantha

Epifani, M.

Eremin, Sergei

Erhart, Jiri

Ersoy, Cem

Eshagh, M.

Eskofier, Bjoern

Esposito, Christian

Esquerre, Carlos

Euser, Tijmen

Evans, Damian

Ewart, P.

Fabbri, Ricardo

Fabrizio, Mazzetto

Fafilek, Günter

Faia, P.M.

Faifer, Marco

Faigl, Jan

Faist, Jerome

Falco, Gianluca

Fan, Pingyi

Fang, Jiancheng

Fang, Qiyin

Fang, Xiaosheng

Fang, Ye

Farahmand, Farid

Farasyn, Andre

Faravelli, Lucia

Farinella, Giovanni Maria

Farooq, Aamir Farooq

Fassi, Irene

Faust, Oliver

Favali, Paolo

Favero, Gabriele

Fazenda, Bruno

Fechteler, Philipp

Fedder, Gary K.

Federolf, Peter

Feemster, Matthew G.

Fei, Juntao

Feller, Jean-Francois

Feller, Karl-Heinz

Feng, Hua

Feng, Jun

Feng, Peter

Feng, Shao-Jun

Feng, Wen-jiang

Feng, Xin

Feng, Yue

Feng, Zhi Hua

Fens, Niki

Fensli, Rune

Fenye, Bao

Fergani, Soheib

Fergus, Jeffrey W.

Fernandes, Fábio

Fernandes, Rohan

Fernández-Caballero, Antonio

Fernandez-Llatas, Carlos

Fernández, Alberto

Fernandez, Fatima

Ferrari, Giorgio

Ferrari, Paolo

Ferrec, Yann

Ferreira, Pedro M.

Ferruz, Joquín

Figueiras-Vidal, Aníbal R.

Filipe Costa, Manuel

Filippi, Anthony

Finke, Stefan

Finocchio, Giovanni

Fischer, Horst

Fischer, Wolf-Joachim J.

Fisher, G. Burch

Flandre, Denis

Fleischer, Jürgen

Fletcher, Mary

Fletcher, Richard

Fleyeh, Hasan

Flint, James

Fodor, Viktoria

Fogue, Manuel

Fojta, Miroslav

Fokoué, Ernest

Fong Yau Li, Sam

Fong, Louis

Fonollosa, Jordi

Font, Enrique

Fontana, Marco

Ford, Andria

Ford, Jason

Foresti, Gl

Forney, Charles F.

Fossati, Andrea

Fotiadis, Dimitrios

Fraga, M. A.

Fragkiadakis, Alexandros

Fraz, Moazam

Fregonese, Luigi

Frense, Dieter

Friedland, Gerald

Friend, James

Frischholz, Robert

Fritze, Holger

Fruk, Ljiljana

Fu, Yue Jun

Fu, Li-chen

Fu, Qiuyun

Fu, Richard

Fu, Tat

Fu, Yu

Fu, Zhifeng

Fujii, Hodaka

Fujii, Yoshiaki

Fujikado, T.

Fujioka, Kouki

Fukatsu, Tokihiro

Fung, Carol

Funston, Alison

Furdea, Adrian

Furferi, Rocco

Fusina, Luciano

Gaeta, Matteo

Gagliardi, Gianluca

Gagnol, Vincent

Galán-Vidal, Carlos

GALANTE, Angelo

Galar, Mikel

Galbally, Javier

Galezowska, Joanna

Galgaro, Antonio

Galiana-Merino, Juan Jose

Gallardo-Velazquez, Tzayhrí

Gallay, Michal

Gallimard, Laurent

Gallo, Alessandro

Gamba, Mirko

Gamboa, Hugo

Gamet, Philippe

Gamo, Javier

Ganchev, Todor

Gao, Feng

Gao, Xiaodan

Gao, Yanbo

García Zapirain, Begoña

García-Fandiño, Rebeca

García-Rupérez, Jaime

Garcia-Sanchez, Antonio-Javier

Garcia-Sanchez, Felipe

García-Sánchez, P.

García, Carmelo

Garcia, Cecilia

García, J.A.

Garcia, Jesus

Garcia, Juan Carlos

Garcia, Luis Guilherme Uzeda

Garcia, Nicolas

Garduño-Wilches, I.

Gargiulo, Gaetano D

Garratt, Matt

Garrido, José Luis

Gatesman, Andrew J.

Gaudiuso, Rosalba

Gaulton, R.

Gautier, Matthieu

Gavras, Anastasius

Gaydecki, Patrick

Gaze, David C.

Ge, Maorong

Geddes, Donna T.

Geddis, Demetris L.

Geihs, Kurt

Geissinger, Peter

Geng, Jianghui

Georgiev, Peter

Georgieva, Elena

Georgios, Kambourakis

Gérard, Lachapelle

Gernaey, Krist V.

Geschwindner, Stefan

Ghafar-Zadeh, Ebrahim

Ghayesh, Mergen H.

Ghisi, Aldo

Giacomini, Gabriele

Gibert, Karina

Gierak, Jacques

Gietzelt, Matthias

Gijsen, Frank

Gil, Emilio

Gilad, Assaf A.

Gillen, Greg

Gilmartin, Niamh

Ginart, A.

Gini, Giuseppina

Giorgetti, Andrea

Giouroudi, Ioanna

Giri, Shibashish

Girotto, Emerson Marcelo

Girousi, Stella

Gislum, Rene

Giulia, Volpe

Giusti, Elisa

Glardon, Rémy

Glavin, Martin

Glennie, Craig

Glentis, George-Othon

Glisic, Branko

Glover, Ian A.

Glover, Paul

Goda, Tatsuro

Goffart, Jean-Pierre

Goh, Shu Ting

Goldthorpe, Irene

Golen, Erik F.

Gollmann, Dieter

Goluch, Edgar D.

Golzarian, Mahmood

Gómez Mancilla, Julio César

Gonçalves, Elsa

Gong, Myoung-seon

Gong, Shi Wei

Gong, Xingao

Gong, Yandong

Gonzále, Ramón

Gonzales Rodriguez, José

Gonzalez Benecke, Carlos Alberto

Gonzalez Ruiz, Vicente

Gonzalez, Joaquin

Goodwin, Jeffrey

Goodwin, Thomas

Goodyer, Eric

Goonetilleke, Ravindra

Goossen, Keith

Gordon, Jeffrey M.

Gotoh, Kazuma

Gottron, Christian

Goulet, James-A.

Gouton, Pierre

Graham, Simon J.

Gramaglia, Marco

Granado, Otoniel Lopez

Grandner, Michael A.

Granell Canut, Carlos

Granger, Michael

Grassi, Marco

Grau, Antoni

Gravenkamp, Hauke

Gravina, Raffaele

Gregory, James W.

Greisen, Pierre

Grena, Roberto

Greswell, Ricahrd

Greve, David

Grewal, Gurtej Singh

Griffin, Benjamin A

Griffin, Lisa

Grill, Roman

Grimm, Hartmut

Gröger, Gerhard

Groot, Paul

Groves, Paul

Guan, Haiyan

Guan, Yu

Gubbi, Jayavardhana

Gubitta, Andrea

Güemes, Alfredo

Guerrero-Castillanos, Fermi

Guerrero, Cesar

Guerrero, Josechu

Gueundelman, Eran

Guidetti, Riccardo

Guillevic, Pierre

Guillier, Maude

Guimarães, A.o.

Guinaldo, María

Guivant, Jose

Guo, Huimin

Guo, Jiantao

Guo, Lei

Guo, Qinghua

Guo, Shirui

Guo, Tuan

Guo, Xulin

Guo, Ying

Gupta, Maneesh

Guthausen, Gisela

Gutierrez-Blanco, Oscar

Gutiérrez-Capitán, Manuel

Gutierrez, Abelardo

Gutiérrez, Daniel

Guy, Chris

Guzman, Salvador Cobos

Gwin, Joseph T.

Ha, Sangtae

de Haan, Victor-Otto

Habekost, Martin

Haber-Pohlmeier, Sabina

Hager, Martin D.

Hahn, Guenter

Haick, Hossam

Haindl, Michal

Hall, David

Hall, Drew A.

Hallez, Hans

Hamada, Nozomu

Hambly, Adam

Hammoudeh, Mohammad

Han, Eun Jin

Han, Jae-hung

Han, Joon Hee

Han, Song

Han, Zhenjun

Hands, Philip

Hanna-Pladdy, Brenda

Hara, Motoaki

Harbron, Elizabeth J.

Harding, Celia

Harle, Robert

Harper, Jason C.

Harren, F.j.m.

Harrison, Andrew

Hartnagel, Hans

Harvey, David M.

Hashemian, H.M.

Hassan, Mohammad Mehedi

Hassanpour, Pezhman

Haufe, Stefan

Häußermann, Angelika

Hautefeuille, Mathieu

Havsgård, Geir Bjarte

Hawkes, Jeremy

Hayazawa, Norihiko

Hayes, Monson H.

Hayward, Ryan C.

He, David

He, Jr-hau

He, Ran

He, Xuefeng

He, Zaixing

Healy, William M.

Heathers, James A.J.

Heinrich, Mattias P.

Heisey, Curtis W.

Heiskanen, Arto

Heleno, Sandra

Hellbrück, Horst

Hellen, Edward

Helms, John

Henderson, Robert

Heng, Sabrina

Henry Chapman, Henry

Hensel, Oliver

Hentschel, Mario

Heo, Junyoung

Her, Shiuh-chuan

Hernández-Pajares, Manuel

Hernandez-Ros, Claudio Aroca

Hernández, Francisco

Hernández, Luis

Hernandez, Victor

Herrera-May, A. L.

Herrera, E. Pulido

Herrera, Rafael Morales

Hertlein, Heinz

Hiak, Tibor

Higgins, Chad

Hilaire, Thibault

Hilker, Thomas

Hiller, Karsten

Hillier, Andrew C.

Hinders, Mark

Hiromori, Akihito

Hirose, Kiyoshi

Hiroshi P., Sato

Hirsch, Thomas

Ho Kang, Byeong

Ho, Jiun-huei

Ho, Johnny Chung Yin

Ho, Wang-Hei

Hocke, Klemens

Hodgkinson, Jane

Höfling, Sven

Hofmann, Martin

Hofmann, Ulrich G

Holland, Stephen D.

Hollinger, Geoffrey

Holmes-Smith, A. Sheila

Honda, Kiyoshi

Honeychurch, Kevin

Hong, Bonghee

Hong, Men

Hong, Minghui

Hong, Seong-Hyeon

Hong, Xiaopeng

Hoof, Chris Van

Hopfgartner, Frank

Horauer, Martin

Hori, Maiya

Horita, Tadayoshi

Hornak, Joseph P.

Horng, Mong-fong

Horng, Shi-jinn

Horobin, Richard W

Horsch, Alexander

Horvath, György

Hoshino, Kazunori

Hossain, A.k.m. Mahtab

Hossny, Mohammed

Hou, Liqun

Hou, Tsung-chin

Howard, Ayanna

Hristoforou, Evangelos

Hsieh, Chih Cheng

Hsu, Chien-chang

Hsu, Yeh-Liang

Hsu, Yi-Chu

Hu, Chao

Hu, Hongxin

Hu, Jiankun

Hu, Ning

Hu, Pingan

Hu, Sheng

Hu, WeiHua

Hu, Xiaogong

Hu, Yong

Hu, Yongjian

Hu, Yuh-Chung

Hua, Hui Guo

Huang, Bohr-ran

Huang, Cheng-yang

Huang, Chi-hua

Huang, Chih-ching

Huang, Ching-Chun

Huang, Ching-lien

Huang, Chun Jen

Huang, Guangyan

Huang, Jingfang

Huang, Jingyun

Huang, Jinsong

Huang, Liquan

Huang, Shasheng

Huang, Shih-jen

Huang, Wei

Huang, Y

Huang, Yi-Wen

Huang, Ying

Huang, Zhiyao

Huang, Zifang

Huber, Jochen

Hughes, Michael P.

Huh, Do Sung

Huh, Eui Nam

Huitzil, César Torres

Hulle, Marc M. Van

Hung, Jui-chung

Hung, Kuo-yung

Hur, J.

Hussein, Mohamed

Hussein, Walid B.

Huston, Dryver R.

Hutsel, Michael R.

Huynh, Du

Hwang, Han-Jeong

Hwang, Hoyoung

Hwang, Jenq-neng

Hwang, Suk-seung

Hwang, Wen-jyi

Hwang, Yongha

Hwangbo, Myung

Hynynen, Katja M.

Hyodo, Mamoru

Newton, Michael I.

Iakovidis, Dimitris K.

Ibarra-Castanedo, Clemente

Ibrahim, S.a.

Ibrahimi, Khalil

Ichikawa, Shuichi

ieng, siohoi

Iera, Antonio

Ihalainen, Petri

Ikonomidou, Vasiliki N.

Ilyas, Potamitis

Im, Hyungsoon

Imato, Toshihiko

Ince, Gokhan

Ingebrandt, Sven

Innocenti, Bianca

Inoue, Takuro

Ioannis, Antoniadis

Iocchi, Luca

Iqbal, Rahat

Ishida, Hiroshi

Ishihara, Susumu

Ismach, Ariel

Itoh, Tetsuji

Iwami, Kentaro

Iwamoto, Shinichiro

Iyota, Taketoshi

Jafarkhani, Reza

Jaffrezic-Renault, Nicole

Jain, Ankit

Jakubikl, W.P.

Jalkanen, Tero

Jamal, Mamun

Jan, Shau-Shiun

Janagama, Harish

Janeiro, Fernando M.

Jang-Myung, Lee

Jang, Shinae

Jannett, Thomas C.

Jaren, Carmen

Javidi, Bahram

Jayasuriya, Hemantha

Jedryczka, Cezary

Jemielniak, Krzysztof

Jenkins, Gregory S.

Jenn, David

Jeon, Doyoung

Jeon, Jae-wook

Jerschow, Alexej

Ji, Hai-Feng

Ji, Zhen

Jia, Juncheng

Jia, Min

Jia, Xinhua

Jiang, Dong

Jiang, Joe-Air

Jiang, Kaili

Jiang, Ming-Shun

Jiang, Ning

Jiang, Ping

Jiang, Wenbin

Jiang, Yadong

Jiang, Yi

Jiang, Yifei

Jiang, Yueming

Jimenez-Berni, Jose A.

Jiménez, Felipe

Jin, Dayong

Jin, Guoyong

Jin, Lianwen

Jin, Ningde

Jin, Qinghui

Jin, Shuanggen

Jin, Xin

Jing, Xiao Yuan

Jing, Yang

Jing, Zhongliang

Jirka, Simon

John, Vijay

Johnson, Blake

Jones, Barry

Jones, Caroline

Jones, John D.

Jones, Kinzy

Jones, Philip

Jones, Richard D.

Jones, Scott B.

Jones, Steven

Jong, Gwo-jia

Jordan, Kieran

Jorgensen, Palle

Joseph, Dileepan

Joshi, Rakesh Kumar

Joubert, S. V.

Jouffrais, C.

Jugo, Josu

Juhlin, Christopher

Juliá, Miguel

Jung, Tzyy-ping

Jung, Yeonwoong

Jungnickel, Volker

Junkwon, Phorntipha

Jwo, Dah-Jing

Kaasalainen, Sanna

Kada, Martin

Kahn, H.

Kaigala, Govind V.

Kaitovic, Jelena

Kakugawa, Hirotsugu

Kalantar Zadeh, Kourosh

Kalantar-zadeh, Kourosh

Kalcher, Kurt

Kaltsas, Grigoris

Kamboh, Awais

Kamikouch, Azusa

Kamsu-Foguem, Bernard

Kan, Xianwen

Kandori, Akihiko

Kandris, Dionisis

Kanellos, George T.

Kang, Lifeng

Kang, Shin-Won

Kanhere, Salil

Kanoun, Olfa

Kanta, Bera Lakshmi

Kańtoch, Eliasz

Kaplan, Alexander

Kar, Piyush

Karabchevsky, Alina

Karacolak, Tutku

Karczmarek, Paweł

Karim, Karim S.

Karimi, Hamid Reza

Karnstedt, Marcel

Karp, Matti

Kataoka, Takashi

Katsaros, Dimitrios

Katsis, C.D.

Katz, Brian F.G.

Kaur, Sumanjeet

Kavehrad, Mohsen

Kealy, Allison

Kedzierski, Michal

Keegan, Neil

Keenan, D. Barry

Keller, Stephan Sylvest

Keller, Thomas

Kemper, Björn

Kennedy, Hugh L.

Ker, Jun-ing

Kerman, Kagan

Kesler, Shelli

Khaliulin, Igor

Khan, Asiya

Khan, Mohidus Samad

Khan, Samee U.

Khan, Shafiullah

Khirallah, Kareem

Khoshmanesh, Khashayar

Khot, Lav R.

Khushaba, Rami

Kibler, Bertrand

Kiefer, Peter

Kim, Beomjoon

Kim, Bok-Hyun

Kim, Bongsoo

Kim, Changhoon

Kim, Do Hyun

Kim, Dong-Eun

Kim, Dong-sung

Kim, Dong-yoon

Kim, Euntai

Kim, Hak-Sung

Kim, Hee-Cheol

Kim, Hong-Seok

Kim, Hyung Joo

Kim, Ig-Jae

Kim, Jae Chang

Kim, Jaehwan

Kim, Jaeyoun

Kim, Jaihie

Kim, Jung-Ha

Kim, Jungsuk

Kim, Ki-bok

Kim, Ki-Hyun

Kim, Kihyun

Kim, Kwang Jin

Kim, Kwang S.

Kim, Moon S.

Kim, Moonseong

Kim, Mooyoung

Kim, Sangchoon

Kim, Se-heon

Kim, Seon Joo

Kim, Seung Keun

Kim, Sohee

Kim, Sung Jin

Kim, Sunwook

Kim, Uksun

Kim, Yeesock

Kim, Yong

Kim, Young Ok

Kimachi, Akira

Kinnunen, Matti

Kinnunen, Teemu

Kintzios, Spiridon

Kinyua, Johnson D. M.

Kirby, Jon

Kirchgessner, Norbert

Kirk, Katherine

Kirkko-Jaakkola, Martti

Kirsanov, Dmitry

Kiryati, Nahum

Kisternaya, Margarita

Kivistö, Rauni

Kizek, Rene

Klaoudatou, Eleni

Klatzky, Roberta

Klingbeil, Lasse

Klous, Miriam

Knechtel, R.

Kneip, Laurent

Knez, Mato

Knobbe, Arno

Ko, Chia-Nan

Ko, Seung Hwan

Kobarg, Jan Hendrik

Kobayashi, Kazuki

Koch, Alexander Walte

Koch, Cosima

Kocifaj, Miroslav

Kockro, Ralf A.

Koh, Jaehan

Kolagani, Rajeswari M.

Kolasinski, Kurt W.

Kolk, Arend

Kolpashchikov, Dmitry

Komine, Hidehiko

Kong, Matthew C. R.

Kong, Wanzeng

Konopsky, Valery N.

Koppal, Sanjeev

Kordás, Krisztián

Kosel, Jürgen

Kosma, Kyriaki

Kosmopoulos, Dimitrios

Kostek, Bożena

Kotru, Sushma

Kou, Bao-Quan

Koulen, Peter

Kovačič, Stanislav

Kraut, Nadine

Krejcar, Ondrej

Krishnan, Subramanian

Kritten, Lena

Krolak, Aleksandra

Kropelnicki, P

Krstajić, Nikola

Krusienski, Dean

Ktena, Aphrodite

Kuang, Boyan

Kuang, Kevin

Kubby, Joel

Kubiak, Krzysztof J

Kuchel, Philip

Kudelski, Michal

Kulathumani, Vinod

Kulvietis, Genadijus

Kumar, Neelesh

Kumar, Pradeep

Kumar, Vijay

Kuochih, Chuang

Kurabayashi, Katsuo

Kurkuri, Mahaveer

Kuroda, Akio

Kurz, Tobias H.

Kutyniok, Gitta

Kuwahara, Masayasu

Kwak, Moon K.

Kwok, Ngai Ming

Kwon, Dae

Kwon, Il-bum

Kwon, Yongjin

Labiris, Renee

Labruyère, Rob

Lacina, Karel

Ladani, Leila

Laflamme, Simon

Lagkas, Thomas

Lago, Claudimir Lucio Do

Lai, Chin-Feng

Lai, Daniel

Lai, Yongjun

Lakemond, R

Lakemond, Ruan

Lal, Bhajan

Lallart, Mickael

Lam, Hung

Lam, Timothy

Lamberti, Fabrizio

Lambie, Suzanne

Lammers, Peter Schulze

Lamonaca, F.

Lan, Chao-Chieh

Lan, Kun-Chan

Lancry, Matthieu

Landers, Andrew

Lane, Nic

Lang, Hans Peter

Lang, Yin H.

Langer, Markus

Lanzola, Giordano

Laracca, Marco

Larger, Laurent

Laroche, Thierry

Larson, Eric C

Lasaponara, Rosa

Lasky, Ty

Latifi, Hamid

Latka, Ines

Lau, Kin-tak

Lau, Sian Lun

Laurell, Thomas

Laurenzis, Martin

Lay-Ekuakille, Aimé

Lay-Ekuakille, Aime'

Lazar, Cosmin

Lazarus, Nathan

Le-Phuoc, Dahn

Le, Qiang

Leach, Richard K

Leboulanger, Christophe

Leccese, Fabio

Leckie, Chris

Lee-Thedieck, Cornelia

Lee, Byeong Ha

Lee, Byoungho

Lee, Caroline S.

Lee, Chan-Su

Lee, Chang-Gun

Lee, Chao-hsien

Lee, Chen-yi

Lee, Cheng-Chi

Lee, Cheng-Kang

Lee, Chengkuo

Lee, Chi-Yuan

Lee, Chia-Yen

Lee, Ching-Ting

Lee, Dong-ryul

Lee, Learn Han

Lee, E. Stanley

Lee, Eui Chul

Lee, Gyu Myoung

Lee, Hian Kee

Lee, Howard

Lee, Hsiang-Chieh

Lee, Huang-chen

Lee, Hungkyu

Lee, Jay

Lee, JB

Lee, Jen-chun

Lee, Jinyi

Lee, Jong-heun

Lee, Jong-Lam

Lee, Jongjae

Lee, Jung Keun

Lee, Keekeun

Lee, Moon Gu

Lee, MunKyu

Lee, Sang-Suk

Lee, Seung-Woo

Lee, Seungchul

Lee, Shie-jue

Lee, Sukhan

Lee, Sungyoung

Lee, Tim K

Lee, Winson C.C.

Lee, Ya-Ju

Lee, Yi-kuen

Lee, Yongkoo

Lee, Young Hoon

Lee, Young-Koo

Legrand, Nicolas

Lei, Chi-Un

Lei, Chong

Lei, Thomas K. F.

Lei, Yinjie

Leicht, Lennart

Leiman, Peter

Leis, John

Leisner, Madeleine

Leman, Marc

Lemmon, Michael

Lemoyne, Robert

Lenzen, Christoph

Leone, Alessandro

Leonov, Vladimir

Léopoldès, J.

Lera, Francisco

Lerasle, Frédéric

Lerma, Jose L.

Leutenegger, Marcel

Levashova, Tatiana

Lewis, Grace

Li, Baopu

Li, Bing

Li, Bofeng

Li, Changzhi

Li, Chunming

Li, David Minghui

Li, Debiao

Li, Deren

Li, Dongmei

Li, Dongsheng

Li, Fei

Li, Gao-ren

LI, Genxi

Li, Haiyun

Li, Hao

Li, Jun

Li, Junhong

Li, Qingli

Li, Shuai

Li, Wei

Li, Wen J.

Li, Wenzhong

Li, Xi

Li, Xiaoli

Li, Xin

Li, Xin Xin

Li, Yongxin

Li, Yundong

Li, Yuting

Lian, Feng-Li

Liang, Chiu-kuo

Liang, Dawei

Liang, Dong

Liang, Hong

Liang, Junrui

Liao, Hongen

Liao, Lun-de

Liao, Shaolin

Liao, Shengcai

Liao, Yi

Liarokapis, Fotis

Liberles, Stephen D.

Lieberzeit, Peter

Lii, Keh-shin

Lim, Chae Hoon

Lim, Kok-Sing

Lim, Mayasari

Lim, Ming

Lim, Seokbin

Lim, Yah Leng

Limão-Vieira, P

Limberger, Hans Georg

Lin, Bin

Lin, Bor-Shyh

Lin, Chii-Wann

Lin, Chin-feng

Lin, Ching-Po

Lin, Fang

Lin, Feng

Lin, Jing

Lin, Jzau Sgeng

Lin, Pei-chun

Lin, Shan

Lin, Sidney

Lin, Wei

Lin, Weiyao

Lin, Yirong

Lin, Yu-shiang

Lin, Yue-Der

Linden, Maria

Lingua, Andrea

Lissek, Hervé

Liu, Chuanjun

Liu, Er

Liu, Fangde

Liu, Fengquan

Liu, Hongbin

Liu, J. Z

Liu, Jian

Liu, Jianlin

Liu, Jie

Liu, Jiemin

Liu, Jing

Liu, Jingbin

Liu, Ju

Liu, Li

Liu, Lusan

Liu, Peixiang

Liu, Peter

Liu, Sisi

Liu, Tao

Liu, Tsu-Jae King

Liu, Xiaofeng

Liu, Xiaohe

Liu, Xinyu

LIU, Yanjun

Liu, Yipeng

Liu, Zhong

Llobet, E.

Llor, Jesus

Llorens Calvedas, Jordi

Lo, Anthony

Lo, Chien-fong

Lo, Sherman

Lochman, Steffen

Loh, Kenneth J.

Lohan, Elena-simona

Loia, Vincenzo

Lojou, Elisabeth

Loke, Seng W.

Lomer, Mauro

Lon A., Wang

Longo, Domenico

Lonini, Luca

Loock, Hans-Peter

Lopes, Waslon Terllizzie Araujo

Lopez-Amo, Manuel

López-de-Ipiña, Diego

López-Rubio, Ezequiel

Lopez-Salcedo, Jose A.

Lopez-Vetrone, Sylvia

Lopez, Carlos

Lopez, Joaquín

Lopez, Juan

López, Mariano

López, Tezozomoc Pérez

Lorefice, Salvatore

Lorenz, Michael G.

Lorenzani, Emilio

Lotfi, Chaari

Lou, Xinhui

Lou, Xizhong

Louarn, Guy

Loui, A.

Loutas, Theodoros

Loutfi, Amy

Love Jr., Norman

Lovisolo, Lisandro

Lu, Chia-Liang

Lu, Fa-Ke Frank

Lu, Ge Yu

Lu, Ge-Yu

Lu, Jian

Lu, Jiwen

Lu, Ming-chyuan

Lu, Peter J.

Lu, Ye

Lu, Yuangang

Lu, Zongqing

Luches, Armando

Lucieer, Arko

Lück, Erika

Luckarift, Heather R.

Lucklum, Ralf

Ludwig, Nicholas G.

Ludwig, Roland

Ludwig, Sonja C.

Lufaso, Michael

Luján, Jose Luis Poza

Luna, A.S.

Luo, Ching-Hsing

Luo, Jianwen

Luo, Wei

Luximon, Ameersing

Luyckx, Geert

Lv, Yi

Lyle, Stacey D.

Ma, Bin

Ma, Ning

Ma, Qiulin

Ma, Shaodan

Ma, Yong

Mabuchi, Mamoru

Macagnano, A.

Macek, Jan

Machado, Sergio A.s.

Machida, Masashi

Mackay, Ruth

MacMillan, Bryce

MacNaughton, Sam

Maddalena, Luca

Madeira, Sergio

Maeda, Isamu

Maehle, Erik

Maeng, Sunglyul

Maetzler, Walter

Magda, Jules J.

Magliulo, Maria

Mahlein, Anne-Katrin

Maier, Robert R. J.

Maio, Francesco Di

Mak, Peng Un

Makarov, Denys

Makinwa, Kofi A. A.

Malekian, Reza

Mallick, Pankaj

Malmendal, Anders

Malocha, Don

Mamun, Quazi

Man, Hong

Manamanni, Noureddine

Mancini, Adriano

Mancini, Clara

Mander, John

Mandic, Danilo P.

Manes, Gianfranco

Mangina, Leni

Manickam, Arun

Manikas, Athanassios

Manini, Todd M

Mankin, R. W.

Mankodiya, Kunal

Manome, Yoshinobu

Mansfield, Jessica

Mansurova, Svetlana

Marasco, Emanuela

Marashdeh, Qussai

Marcos, Jorge

Mariani, Stefano

Marinesco, Stéphane

Mariscotti, Andrea

Märk, Tilmann D.

Marken, Frank

Marković, Ivan

Marks, R.S.

Marnane, William

Marnet, Pierre-Guy

Marpu, Prashanth Reddy

Marques, Paula A.A.P.

Marquez Barja, Johann

Mars, Jérôme

Martalo, M.

Martalò, Marco

Martelli, Francesca

Martin, Angel

Martin, Hans

Martin, Sarah

Martinez Dominguez, Francisco

Martínez Durá, Rafael J.

Martínez Rosas, Miguel Enrique

Martinez-Huitle, Carlos

Martínez-Tomás, Rafael

Martinez, Fernando Seoane

Martínez, José F.

Martins, Hugo

Martinsanz, Gonzalo

Martinuzzi, Sebastian

Marvin, Jonathan

Marzani, Alessandro

Marzin, Giovanni

Masamune, Ken

Masiero, Andrea

Maslen, Eric Harvey

Masot, Rafael

Massai, Rossano

Massanet, Sebastia

Masud, Mehedi

Mather, Melissa

Mathews, Sunish

Matias, Ignacio R.

Matko, Vojko

Matos, Aníbal

Matsuda, Yu

Maurits, Natasha M.

May-Arrioja, Daniel A.

Mayer, Dirk

Mayhew, Christopher

Mazzei, Luca

Mazzini, Gianluca

Mazzolai, Barbara

Mazzotta, Elisabetta

McCarty, Owen

McEvoy, James

Mcgibbon, Chris

McGrath, Denise

McNeary, Rory

McNeil, Melanie

Medrano, Hipolito

Medwell, Paul

Meek, Sanford G.

Mejías Alvarez, Luis

Mellis, Craig

Mellone, Sabato

Melo, POS Vaz de

Melodia, Tommaso

Melzak, Kathryn

Memis, Omer Gokalp

Méndez Zorrilla, Amaia

Meng, Hongying

Meng, Lim Kian

Meng, Ling

Menna, Fabio

Menzel, Andreas

Mera, David

Meratnia, Nirvana

Merkx, Maarten

Merletti, Roberto

Merritt, Matthew

Mertz, Christoph

Mery, Domingo

Meschede, Dieter

Meszaros, Tamas

Metze, Konradin

Mi, Yongli

Mialon, Arnaud

Miao, Jianmin

Miao, Yinping

Michalek, Maciej

Michałowski, Tadeusz

Michalska, Agata

michelotti, francesco

Mignani, Anna

Miguel, Leandro Fleck Fadel

Mikada, Hitoshi

Miki, Hirofumi

Milan, David

Milekic, Slavko

Milicchio, Franco

Millan Almaraz, Jesus Roberto

Miller, Jimmie

Mills, Steven

Miluzzo, Emiliano

Miñambres Marcos, Víctor Manuel

Minardo, Aldo

Mingesz, Robert

Minor, Mark Andrew

Mirapeix, Jesus

Mirsky, Vladimir

Mishra, Deepak R.

Misra, Manoranjan

Misron, Norhisam

Mitchell, Paul

Mitrou, Nikolas

Mitsantisuk, Chowarit

Mittlboeck, Manfred

Miura, Norio

Miura, Tomoaki

Miyagishi, Makoto

Miyake, Shiro

Miyamori, Yasunori

Mizsei, János

Mizutani, Yoshihiro

Mkandawire, Martin

Modotto, Daniele

Moewes, Christian

Moghadam, Peyman

Moh, Sangman

Mohammed, Samer

Mohemmed, Ammar W.

Möhwald, Helmuth

Monakhova, Yulia

Monroy, Javier González

Monserrat, Oriol

Montalvo, Idel

Monteiro, João

Monteiro, Miguel P.

Montereali, M.R.

Montes, Juan

Montewka, Jakub

Monzón-Hernández, David

Moon, Doo Kyung

Moon, In-Kyu

Moon, Jong-yun

Moore, Edward

Moore, Paul

Morell, Gerardo

Moreno, Daniel

Moreno, Luis

Morgan, Elise F.

Moritz, Chet T.

Morozov, Maxim

Morrin, Aoife

Morris, Robert H

Morton, Peter

Moshirfar, Majid

Moshou, Dimitrios

Moshou, Dimitris

Mosiello, Lucia

Moslehi, Behzad

Mossi, Karla

Mou, Chengbo

Mouaziz, Schahrazede

Mouftah, Hussein T.

Moyne, James

Mrad, Nezih

Mucherino, Antonio

Muena, J.p.

Mühl, Gero

Mujika, Maite

Mukherjee, Joyeeta Mitra

Mukherjee, Partha Sarathi

Mukhopadhyay, Subhas Chandra

Mulholland, Tony

Muller, Cobus

Munafò, Andrea

Muñoz-Gea, Juan Pedro

Muñoz, Diego Ramírez

Munsell, Brent C.

Muraleedharan, Rajani

Murata, Masayuki

Murillo, Carmona Javier

Murimi, Renita

Musameh, Mustafa

Musić, Josip

Mussetta, Marco

Mustafa, Ghulam

Musteata, Marcel

Myers, Oliver J.

Na'es, Maram

Nabki, Frederic

Nadarajah, Nandakumaran

Naemi, Roozbeh

Nagahara, Masaaki

Nagatani, Naoki

Nagerl, Valentin

Nagy, Attila

Najafi, Nader

Nakagawa, Elisa Y.

Nakamura, Daisuke

Nakamura, Hideaki

Nakamura, Kentaro

Nakamura, Masatoshi

Nakano, Kimihiko

Nakata, Munetaka

Nakayama, Masashi

Nakazawa, Atsushi

Nam, Boohyun

Nanayakkara, Thrishantha

Nanni, Loris

Nansen, Christian

Nappi, Michele

Narayan, Roger

Narberhaus, Franz

Nascetti, Andrea

Nascimento, Sergio

Nassar, Sameh

Nayak, Kali

Naylor, Graham

Ndieyira, Joseph

Nedelchev, Lian

Nedevschi, Sergiu

Neethirajan, Suresh

Negrão, Cezar O.R.

Neri, Giovanni

Nerry, Françoise

Neska, Anne

Neto, P.

Neudeck, Philip

Neuman, Michael R.

Neves, Paulo A.C.S.

Newhook, John P.

Newman, Kimberly E.

Ng, Joseph

Nguyen, Thanh Phuong

Ni, Bingbing

Ni, Yongnian

Niazi, Javed H.

NICOLA, Mario

Niederleithinger, Ernst

Nieminen, Tuukka

Niemz, Peter

Nieto-Taladriz, Octavio

Nigris, De

Niimi, Tomohide

Niklaus, Frank

Nikolopoulos, Dimitrios

Niku, Saeed

Nimmermark, Sven

Nishihara, Akinori

Nitzan, Meir

Niu, Chen

Niu, Qunfeng

Niu, Xiaoji

Nobes, David S.

Noessner, Jan

Nogueira, Rogério N.

Nordlander, Peter

Norman, Kent

Norton, Tomas

Noureldin, Aboelmagd

Novak, Domen

Novella, José Ignacio Moreno

Nowotny, Thomas

Nunziata, Ferdinando

Nuske, Stephen

Nyka, Krzysztof

O'byrne, Sean

O'Grady, Michael

O'neill, Hugh

O'shaughnessy, Patrick T.

O'Donnell, Jodi L.

O'Mahony, Conor

Oberhammer, Joachim

Ocaña, Manuel Miguel

Ochoa, Sergio

Oechsner, Simon

Ogunjemiyo, Segun O.

Ogurtsov, Vladimir I.

Oh, Ilkwon

Ohodnicki, Paul R.

Ohta, Aaron T.

Ohta, Hiromitsu

Okabayashi, Tohru

Okabe, Yoji

Okiror, Grace P.

Oldeland, Jens

Oldham, Keith B.

Olesen, Folke

Olivares, Teresa

Oliver, Gabriel

Olivier-Klein, Jacques

Olmos, A. Martínez

Olmos, Pablo

Olsen, Rasmus

Olsson, Håkan

Olthuis, W.

Olyaee, Saeed

Oprea, Alexandru

Ordonez, Camilo

Orellana, Guillermo

Osaka, Tetsuya

Osborne, R.

Ou, Jian Zhen

Ou, Shumao

Overstraeten, Mme Nancy Van

Oyama, Munetaka

Ozawa, Ryuta

Ozbulut, Osman E.

Ozeki, Yasuyuki

Ozturk, Z.z.

Pacholski, Claudia

Paek, Jeongyeup

Page, Wyatt

Pagiatakis, Spiros

Paixão, Thiago R.l.c.

Pal, Sudeshna

Palacin, Jordi

Palacios, Antonio

Palaniswami, Marimuthu

PALMIERI, Luca

Palombo, Enzo A.

Pan, Jae-Kyung

Pan, Jeng-Shyang

Pan, Meng-shiuan

Pan, Zengxi

Panahandeh, Ghazaleh

Pandey, Gaurav

Panfilis, Simone De.

Pang, Ai-Chun

Pang, Chee Khiang

Pang, Poh Choo

Pang, Jing

Paniagua, Guillermo

Panigrahi, Bighnaraj

Pankanti, Sharath

Pantea, Cristian

Pantelopoulos, Alexandros

Pao, Tsang-Long

Paolesse, Roberto

Papadopoulou, Elena V. M.

Papaelias, Mayorkinos

Pardo, Antonio

Parenthoen, Marc

Parigi, Giacomo

Parisio, Alessandra

Park, Chan Gook

Park, H. S.

Park, Jae Yeong

Park, Jaebyung

Park, Kang Ryoung

Park, Ki-woong

Park, Mi Kyoung

Park, Simon S.

Park, Tae Jung

Park, Yon-Kyu

Park, Yong-Lae

Parmiggiani, Alberto

Passian, Ali

Pastre, Marc

Patias, Petros

Patron-Perez, Alonso

Patterson, Adriana

Pattichis, Marios S.

Pavelko, Roman

Pazderski, Dariusz

Pearson, Matthew R.

Pedersini, Federico

Peham, Christian

Pejcic, Bobby

Pelivanov, I. M.

Pellerin, Christian

Peltoniemi, Jouni

Peng, Hanjng

Peng, Peng

Peng, Peng-Chun

Peng, Yang

Pennacchi, Paolo

Perales, Fco J.

Pereira-Barretto, Marcos R.

Pereira, Alejandro

Pereira, António

Pereira, Carlos Eduardo

Pereira, Emiliano

Pereira, Fabíola Manhas Verbi

Perenzoni, Matteo

Perez Solano, Juan J.

Pérez-López, Briza

Perez, Manuel

Perez, Rodrigo

Peris-López, Pedro

Perkins, Rupert

Perlepes, Spyros P.

Peroulis, Dimitrios

Perrone, Guido

Perumal, Veeramani

Pesatori, Alessandro

Pescaru, Dan

Pesce, Maurizio

Pesci, Arianna

Pessin, Gustavo

Peter, Chong Han Joo

Peter, Van Der Wal

Petit, Nicolas

Petri, Dario

Petroccia, Roberto

Petruzzelli, Vincenzo

Pezoa, Jorge E.

Pezoldt, Jörg

Philippides, Andy

Phillips-Jones, Mary K.

Phothisonothai, Montri

Pianese, Fabio

Piazza, Roberto

Picone, Marco

Piérard, Sébastien

Pierron, Olivier N.

Pignaton de Freitas, Edison

Piikki, Kristin

Piller, Olivier

Pimenta, Tales Cleber

Pimentel, M. Fernanda

PinGang, He

Piras, Marco

Pirotti, Francesco

Pisani, Marco

Pishro-Nik, Hossein

Pissadakis, Stavros

Pista, Angela

Pittman, Todd B.

Piuzzi, Emanuele

Pizzuti, Stefano

Platas-Iglesias, Carlos

Plumeré, Nicolas

Pohanka, Miroslav

Polesel-Maris, Jérôme

Pomalaza-Ráez, Carlos

Ponomareva, Olga N.

Ponzoni, Andrea

Poon, Carmen Chung Yan

Popescu, Gabrel

Porter, Barry

Portillo Rodríguez, Otniel

Posthuma, Truus

Postolache, Octavian

Postylyakov, Oleg V.

Potamianos, Gerasimos G.

Potter, Lee C.

Pottier, Bernard

Powers, David

Prager, Jens

Prakash, Ranganathan

Pratas, Nuno Kiilerich

Prati, Andrea

Prattichizzo, Domenico

Premebida, Cristiano

Preuveneers, Davy

Priha, Outi

Prikhodko, Igor

Pristovsek, Markus

Provine, J.

Prytherch, David

Psimoulis, Panos A.

Pu, Lina

Punning, Andres

Pustan, Marius

Puttonen, Eetu

Pütz, Thomas

Pyk, Pawel

Pyo, Cheol-Sik

Pyun, Jae-chul

Qin, Shi-qiao

Qiu, Jinhao

Qiu, Zhi-cheng

Quandt, Eckhard

Querlioz, Damien

Quero, Jose Manuel

Raahemifar, Kaamran

Radosavljevic, Goran

Radu, Fechete

Radwan, Essam

Rafaja, David

Rajan, Ginu

Rakić, Aleksandar D.

Ramasami, Ponnadurai

Ramirez, Javier Perez

Ramos, Pedro M.

Rana, D.

Ranasinghe, Damith

Randall, Robert B.

Ranieri, Gaetano

Ranji, Mahsa

Ranusawud, M.

Rao, Ashwin

rao, yunjiang

Raquet, John F.

Rasmussen, Morten Dam

Rassaei, Liza

Rattani, Ajita

Rattfält, Linda

Raul, Sanchez-reillo

Rauscher, Alexander

Rawashdeh, Samir A.

Rawat, Danda

Ray, Krishanu

Razionale, Armando

Rea, Giuseppina

Rea, Ilaria

Rebaudengo, Maurizio

Redmond, Stephen

Redondi, Alessandro

Reichl, Tobias

Reid, Alistair

Reig, Càndid

Reiner, Joseph E.

Reinoso, Oscar

Reinstein, Michal

Reis, Manuel J.c.s.

Rekand, T.

Ren, Dahai

Ren, Jimin

Ren, Liyong

Ren, Yong

Ren, Yuan

Rendina, Ivo

Rendón Mancha, Juan Manuel

Rennie, Allan

Reparaz, Juan Sebastian

Reshetilov, Anatoly N.

Reutebuch, Stephen E.

Revsbech, Niels

Reyer, Michael

Rhee, Huinam

Ricciardi, Armando

Rice, Jennifer

Richelli, Anna

Richter, Rolf

Ridgel, Angela

Riedo, Elisa

Riegger, Lutz

Riehm, Mats

Riess, Christian

Rimpiläinen, Ville

Rincón, Fernando

Ripp, Steven A.

Rishpon, Judith

Ritchie, Grant

Rius, Francesc Xavier

Rivera-Martinez, Sonia

Rizvi, Syed

Rizwan, Zahid

Ro, Yong Man

Roatta, Silvestro

Robert, Laurent

Roberts, Dar

Robertson, William M.

Rocca, Paolo

Rocchini, Duccio

Rocha, Luis

Rocha, Luís Alexandre

Rodger, James A.

Rodríguez Ángeles, Alejandr

Rodriguez-Mendez, M.L.

Rodríguez, Antonio Artés

Rodriguez, Francisco J.

Rodriguez, Sergio Alberto

Roeder., Georg

Rogers, John A.

Roggero, Marco

Rogier, Hendrik

Rogowitz, Bernice E.

Rojas, Luis Martin

Roman, Adam

Roman, Chris

Roncagliolo, Pedro A.

Rong, GuoGuang

Ronsse, Renaud

Rosado, Alfredo Muñoz

Rosdi, Bakhtiar

Rosenberger, Christophe

Rosina, Elisabetta

Rossi, Mirco

Rossiter, Jonathan

Rothballer, Michael

Roundy, David

Roundy, Shad

Rousseau, David

Routen, Ash

Roveri, Marco

Rowlands, Alex

Rowley, Howard A.

Rozelle, David M.

Ruan, Haowen

Rubel, Oleg

Rubel, Paul

Rubinsky, Boris

Rubio Solá, Antonio

Rubio-Bollinger, Gabino

Rubio-Largo, A.

Rückert, Ulrich

Rufino, Giancarlo

Ruhl, Henry A.

Ruhlmann, Laurent

Ruiz-Fernández, Luis Ángel

Ruiz-Sandoval, Manuel

Ruizenaar, Marcel

Ruo, Yuan

Ruotsalainen, Laura

Rupitsch, Stefan

Russell, Chris

Ruzgas, Tautgirdas

Ryadnov, Maxim G.

Loutridis, S.J.

Sabatini, Angelo Maria

Sabatini, Silvio

Sabatino, Silvana Di

Sadrpour, Amir

Saeed, Khalid

Saey, Timothy

Safacas, Athanasios

Safina, Gulnara

Sahoo, Prasan Kumar

Sahoo, Satya S

Sakai, Kenshi

Sakaie, Ken E.

Salamone, Salvatore

Salarian, Arash

Salazar-Torres, Jose J.

Salazar, Félix

Salehi, Bahram

Salerno, Marco

Salgado, Andrea Medeiros

Salguero-Chaparro, Lourdes

Sallese, Jean-Michel

Salmon, J.M.

Sammoura, Firas

Sampietro, Daniele

San, Wong Yoke

Sanchez, Jean-baptiste

Sánchez, Jorge Antonio

Sanchez, Luis

Sanchez, Manuel Garcia

Sánchez, María-teresa

Sando, Shinsuke

Sandrin, Todd R.

Sanford, Lindsay

Sang, Shengbo

Sanghoon, Chin

Sanguino, Tomás De J. Mateo

Sanna, Andrea

Sano, Yuji

Sansoni, Giovanna

Santa, José

Santamaria, Amilcare

Francesco

Santana, Pedro

Santiago, Raul

Santonico, Marco

Santos Pérez, Carlos

Santos, Paulo

Santucci, Sandro

Santulli, Carlo

Sanz, J.

Sanza, F.J.

Saponara, Sergio

Sarangan, Venkatesh

Sato, Goro

Satoru, Takahashi

Sausen, Paulo Sérgio

Savo, Salvatore

Sazio, Pier

Sberveglieri, Veronica

Schaap, Iwan A.t.

Schade, Sven

Schaufele, Fred

Schellen, H.l.

Scherer, David R.

Schilt, Stephane

Schindelhauer, Christian

Schindler, Falko

Schipperijn, Jasper

Schlögl, Alois

Schmerr, Lester

Schmid, Thomas

Schmidt, Markus

Schmitt, Katrin

Schmitz, Alexander

Schneiderman, Justin F.

Schödel, René

Schramm, Wolfgang

Schreiber, Fabio A.

Schreve, Kristiaan

Schröder, Tim

Schulze, Holger

Schwödiauer, Reinhard

Scilingo, Enzo Pasquale

Scioscia, Floriano

Scognamiglio, Viviana

Sebastián, José María

Seco-Granados, Gonzalo

Sedlacik, Jan

Seel, Thomas

Seguí, Joan Melià

Segura, José C.

Seguy, Sébastien

Sekercioglu, Ahmet

Semenova, Yuliya

Semke, William

Senesky, Debbie

Sengul, Cigdem

Seo, Sang Soo

Seo, Seong

Serikawa, Seiichi

Serna, Sergio

Serôdio, Carlos

Serranti, Silvia

Serrat, Joan

Setou, Mitsutoshi

Settles, A. Mark

Seung-Bok, Choi

Seyedi, MH

Seys, Stefaan

Sgorbini, Barbara

Shafait, Faisal

Shah, Nilam

Shah, Shishir K.

Shahid, Adnan

Shan, Shiguang

Shapiro, Benjamin

Shaporev, Aleksey

Shapter, Joe G.

Sharafi, Bahar

Sharma, Abishek

Sharma, Mridula

Sharon, Gil

Shemshad, Javad

Shen, Bin

Shen, Ju

Shen, Tsu-Wang

Shen, Wen

Shen, Y.

Shen, Yajing

Shen, Ying

Sheng, Michael

Sheu, Fang-Wen

Shevkoplyas, Sergey S.

Shi, Bertram E.

Shi, Jiancheng

Shi, Peng

Shi, Wei

Shi, Yi-wei

Shiang, Tzyy-Yuang

Shibusawa, S.

Shih, Tzay-farn

Shih, Wen-Pin

Shih, Wendian

Shihada, Basem

Shikida, Mitsuhiro

Shim, Duk-sun

Shim, Joon Hyung

Shimabukuro, Yosio

Shimizu, Ken D.

Shin, Jae Ho

Shin, Kyu-Ho

Shitashima, Kiminori

Shu, Lei

Shu, Xuewen

Shu, Y. C.

Shusterman, Vladimir

Sicari, Sabrina

Sichero, Laura

Sidén, Johan

Siebert, J. Paul

Sigalotti, L. Di G.

Sikka, Pavan

Silva De Oliveira, Romulo

Silva, João

Silvester, Debbie S.

Silvestre-Blanes, Javier

Simani, Silvio

Simic, Milan

Simion, Cristian E.

Simmons, Steve

Simon, Sven

Simonescu, Claudia Maria

Simonov, Mikhail

Singh, Ahluwalia Balpreet

Singh, Dhananjay

Singh, Ranjan

Sironi, Selena

Sisinni, Emilliano

Siska, Filip

Sithamparanathan, Kandeepan

Sixdenier, Fabien

Sjöberg, Jonas

Skarmeta, Antonio

Skierucha, Wojciech M.

Skrzypczyński, Piotr

Sladek, Jerzy

Slavič, Janko

Slobodian, Petr

Sly, David

Šmíd, Radislav

Smietana, Mateusz

Smith, Christopher

Smith, Emily

Smith, Stephen

Smoukov, Stoyan

Smyth, Andrew

Snape, Jamie

Snellen, Mirjam

Snoussi, Hichem

Snyder, Alexandra

Sobol, Robert W.

Sohgawa, Masayuki

Sohn, Honglae

Soldano, Caterina

Soleimani, M.

Soleimanpour, Amir Masoud

Solovchenko, Alexei

Sommer, Gregor

Sommer, Rubem Luis

Son, Hungsun

Son, Soogook

Song, Aiguo

Song, Jifeng

Song, Li

Song, Wenzhan

Song, Yi

Song, Yong-Sang

Sorge, Christoph

Sotiriou, Georgios

Soto Campos, Ignacio

Soto, Fulgencio

Soto, Rogelio

Sousa, Armando Jorge

Souto Rosa, Nelson

Spagnolo, Vincenzo

Spears, Iain

Spencer, Billie F

Spinelli, Francesco

Spodek, Jonathan

Srhoj-Egekher, Vedran

Stacey, M.

Stagni, Rita

Ståhl, Göran

Stanacevic, Milutin

Stanca, Sarmiza Elena

Stansfield, Ben

Stark, Eran

Stasch, Christoph

Stash, Natalia

Št'astný, Jakub

Stavrakas, Ilias

Stavropoulos, Thanos G.

Stavroulakis, Georgios E.

Stechele, Walter

Steenhaut, Ir. Kris

Stefanescu, Dan Mihai

Stefani, Alessio

Stefanini, Fabio

Stegagno, Paolo

Steinberg, Ben Z.

Steinberg, Ivana Murković

Steiner, Rudolf

Stellacci, Anna Maria

Stephani, Henrike

Stern, Helman

Sternberg, Harald

Stetten, George

Stevanovic, Dusan

Sthel, Marcelo Silva

Stiros, Stathis

Stobiecka, Magdalena

Stojanović, Goran

Stojanovic, Radovan

Stojmenovic, Ivan

Stoppacher, Norbert

Stork, Anna

Strelcov, Evgheni

Strzelecki, Michat

Štumberger, Gorazd

Su, Ching-liang

Su, Chuan-Jun

Su, Fei

Su, Ing-Jiunn

Su, Steven

Su, Te-jen

Su, Wenzheng

Suárez, José I.

Subbarao, Kamesh

Subirana, Jaume Sanz

Subramanian, Sridhar

Sugano, Masashi

Sugano, Shigeki

Suh, Young-Su

Sun, Cheng

Sun, Chia-Wei

Sun, Gang

Sun, Guanghui

Sun, Hongjian

Sun, Jianhai

Sun, Qizhen

Sun, Stella

Sun, Wei

Sun, Zhenan

Sun, Zhi

Sun, Zhili

Sundramoorthy, Ashok

Sung, DanKeun

Sung, Guo-ming

Sung, Hyung Jin

Sung, Wen-tsai

Sunkara, Vijaya

Surbeck, Heinz

Suutala, Jaakko

Suzuki, Hodaka

Suzurikawa, Jun

Svir, Irina

Swatantran, Anu

Sweet, Julian

Swillens, Abigail

Szabat, Krzysztof

Szczesna-Iskander, Dorota H

Szwoch, Mariusz

Szypłowska, Agnieszka

T. Kubota, Lauro

Tabib-Azar, Massood

Taghizadeh, Masoud

Takabayashi, Masanori

Takashima, Kazuto

Taki, M.

Taki, Masayasu

Talaba, Doru

Talavage, Thomas M.

Tamás, Levente

Tamayo, Javier

Tan, Jindong

Tan, Liansheng

Tan, Xiaoyang

Tanabe, Takasumi

Tandy, Michael

Tang, Fei

Tang, Hao

Tang, Jie

Tang, Shijun

Tang, Wei

Tang, Zhiyong

Taniguchi, Masateru

Tao, Lei

Tapia, Emmanuel

Tarabini, Marco

Tardif, Pierre Martin

Tavío, María

Taylor, Charles C

Tchrakian, Tigran

Teasdale, Margaret E.

Teixeira, Marcos Fernando De

Souza

Tel-Vered, Ran

Temko, Andriy

Temple-Boyer, P.

Tenbrinck, Daniel

Teng, Jun

Tennina, Stefano

Tepe, Kemal

Teranishi, Yuuichi

Terribile, Fabio

Terzic, Edin

Teunissen, P.J.G.

Thessler, Sirpa

Theuwissen, Albert J. P.

Thomas, Jean-Baptiste

Thompson, J.G.

Thompson, R.B.

Thöny-Meyer, Linda

Thoonen, G.

Thouand, Gerald

Thoury, Mathieu

Thulasiraman, Preetha

Tian, Fei

Tian, Guiyun

Tian, Jie

Tian, Lin

Tian, Yuan

Tichoniuk, Mariusz

Tiddeman, Bernie

Tie, Li

Tigbe, William

Timlin, Jerilyn A.

Tits, Laurent

Tjin, Swee Chuan

Tkac, Jan

Toca-Herrera, José Luis

Toda, Masaya

Todescato, Francesco

Tollefsen, Dag

Tollner, Ernest W.

Tomari, Mohd Razali Md

Tombelli, Sara

Tomkiewicz, Dariusz

Tonezzer, M.

Tong, Daoqin

Tong, Limin

Tong, Yan

Tonidandel, Flavio

Torki, Marwan

Torralba-Silgado, Antonio J.

Torres-Costa, V.

Torres-Gomez, Ismael

Torres, Fernando

Torres, Miguel

Touhafi, Abdellah

Trabue, Steven

Trahanias, Panos

Trajkovski, Klemen Kozmus

Tran-Quang, Vinh

Tran, Binh Q.

Tran, Gia Khanh

Traver Salcedo, Vicente

Travieso González, Carlos Manuel

Travieso-González, Carlos M.

Tredicucci, Alessandro

Trémeau, Alain

Treuillet, Sylvie

Triantafyllopoulou, Dionysia

Triggs, Bill

Trincavelli, Marco

Trindade, Marcelo Areia

Trishchenko, Alexander P.

Tron, Roberto

Trusov, Alexander A.

Tsai, Jia-Lin

Tsai, Meng-Tsan

Tsao, Hen-Wai

Tseng, Kuo-Kun

Tseng, W. L.

Tsenkova, Roumiana

Tsourveloudis, N.

Tsuchiya, Toshiyuki

Tu, Peter

Tuan, Chiu-ching

Tuck, Kellie L.

Tung, Steve

Turchetta, Renato

Turner, Martin

Tutsch, Rainer

Uchikoba, Fumio

Uckelmann, Dieter

Ueda, Toshitsugu

Uehara, Keisuke

Ukida, Hiroyuki

Ukkonen, Leena

Uludag, Suleyman

Umasankar, Yogeswaran

Umeda, Kazunori

Ureña, Jesus

Ursu, Ioan

Urzaiz, Gabriel

Usamentiaga, Rubén

Uslander, Thomas

Uslar, Mathias

Uysal, Ismail

Uyttendaele, Mieke

Vacher, M

Vaisocherová, Hana

Valente, A.

Valenza, Gaetano

Valenzuela, L.

Vallan, Alberto

Vallee-Belisle, Alexis

Valls, Xavier Binefa

Van Belle, Vanya

van Bennekom, W.P.

Van den Noorta, Josien C.

Van Der Aalst, Wil

Van der Meer, Jan Roelof

Van Doren, Steven R.

van Eerdenburg, Frank J C M

van Hees, Vincent

Van Huffel, Katrijn

Van Ommen, J. Ruud

Vanderelst, Dieter

Vann, R.G.L.

Varberg, Thomas

Vargas, Francisco

Varhola, Andrés

Varotsos, C.

Vaseashta, Ashok K.

Vasilakos, Athanasios V.

Vassiliou, Vasos

Vassilis Gikas, Vassilis

Vázquez, Carmen

Vedru, Jüri

Velaga, Nagendra

Velàzquez, Ramiro

Veltink, Peter

Vena, Arnaud

Vergallo, Patrizia

Vergara, Alex

Verhaegen, Michel

Verhagen, Sandra

Verin, Alexander D.

Verma, Dinesh

Vernazza, Gianni

Verrelli, Cristiano Maria

Verrelli, Giorgio

Vettel, Jean

vettore, antonio

Vezzetti, Enrico

Vichi, Francesca

Vidal-Verdu, Fernando

Viegas, Diana

Vieira, Fausto

Vieira, Luiz

Viitala, Tapani

Vilariño, David

Villa, Flavio

Villanueva, Guillermo

Villanueva, Luis Guillermo

Villatoro, Joel

Virk, Gurvinder

Visconti, Fernando

Visser, Arnoud

Visser, Hubregt J.

Viswanathan, Subramanian

Vittuari, Luca

Viulet, Tiberiu

Viventi, Jonathan

Vivier, Vincent

Vlachos, M.

Voiculescu, Ioana

Volden, Sondre

Volinsky, Alex

Volos, Haris I.

Volpi, Nicola

Volz, Jürgen

Vong, Chi-Man

Vrazic, Mario

Vural, Serdar

W. Berge, Therese

Wade, Scott

Waggoner, Alan S.

Wahab, M.A.

Wakui, Shinji

Waldvogel, Siegfried R.

Waller, Jess

Wallhoff, Frank

Walter, Carina

Walter, Thomas R.

Waluyo, Agustinus Borgy

Wan, Kai Tai

Wan, Yan

Wang, Baojun

Wang, Bo

Wang, Chao

Wang, David W.

Wang, Dexiang

Wang, Fei

Wang, Haifeng

Wang, Hailin

Wang, Haoping

Wang, Hongcheng

Wang, Hsiang-Chen

Wang, Huaqing

Wang, Jeen-Shing

Wang, Jiachou

Wang, Jian-neng

Wang, Jianguo

Wang, Jianxin

Wang, Jiao

Wang, Jing

Wang, Jing-Ling

Wang, Kaiying

Wang, Keping

Wang, Lei

Wang, Li-Chun

Wang, Meng C

Wang, Min

Wang, Ming

Wang, Mu-chun

Wang, Pengfei

Wang, Ping

Wang, Qi

Wang, Qinghua

Wang, Shangfei

Wang, Shaopeng

Wang, Shengwei

Wang, Shui-jinn

Wang, Song

Wang, Tao

Wang, Wen-qin

Wang, Wenyi

Wang, Xianqiao

Wang, Xiaona

Wang, Xin

Wang, Xinheng

Wang, Xiping

Wang, Xu

Wang, Xu-dong

Wang, Yan

Wang, Yao-tien

Wang, Yazhou

Wang, Yi-zhong

Wang, Yixin

Wang, Yu-jen

Wang, Yuelin

Wang, Yun

Wang, Yunhong

Wang, Zhe

Wang, Zhenfeng

Wang, Zhenxin

Wang, Zheyao

Wang, Zhiwei

Wang, Zhongyu

Wang, Zinan

Ward, Michael C.L.

Wardencki, Waldemar

Warren-Smith, Stephen

Wartzek, Tobias

Washington Ii, Aaron L.

Wasige, Edward

Wasisto, Hutomo Suryo

Wassertheurer, Siegfried

Watanabe, Eiki

watanabe, tetsuyou

Waterloo, Maarten J.

Watkins, Anthony N

Watson, Stuart

Webster, Don

Weddell, Alex

Wegner, Jan Dirk

Wei, Hung-yu

Wei, Lei

Wei, Xiaolin

Weidemann, Alan

Weigel, Robert

Weimann, Claudius

Weiss, Martin

Weiss, Stephan

Weisz-Patrault, Daniel

Welemane, Hélène

Wen, Chih-yu

Wen, Yean-fu

Wendel, Jan

Weninger, Felix

Wentzloff, David D.

Wermser, Diederich

Werneck, Marcelo

Werner, Jürgen H.

Wetz, David Alan

Wezyk, Piotr

Whang, Thou-jen

Whelan, Robert

White, Daniel

Whitelonis, Nick

Whittell, George

Wiegärtner, Sven

Wielopolski, Lucian

Wiggenhauser, Herbert

Wilder-Smith, Petra

Wiliem, Arnold

Willander, Magnus

Williams, Kathryn R.

Williams, Paul A.

Willis, Pascal

Willmott, Geoff

Willner, Itamar

Willson, Richard

Wilson, Alphus

Winston Su, Wei-Wen

Withayachumnankul, Withawat

Wlodkowic, Donald

Wójcik, Waldemar

Wójcikowski, Marek

Wolffenbuttel, Reinoud

Wolkers, Willem F.

Won, Jong-Hoon

Wong, Danny K. Y.

Wong, K. Thomas

Wong, Kin-hong

Wong, Tak Sing

Wong, Wai-choong

Wood, Stephen L.

Woodhouse, Jim

Wooldridge, Jenny

Work, Paul A.

Wozniak, Adam

Wright, Bill

Wu, Fan

Wu, Hsien-Tsai

Wu, Jiaji

Wu, Jian Xiong

Wu, Judy Z.

Wu, Nan

Wu, Qiang

Wu, Quincy

Wu, Ren-Jang

Wu, Shixin

Wu, Weiwei

Wu, Wenzhuo

Wu, Xiangqian

Wu, Xiaosheng

Wu, Xue-zhong

Wu, Yafeng

Wu, Yifan

Wynendaele, Evelien

Xanthopoulos, Petros

Xi, Juntong

Xia Cao, Xia

Xia, Andong

Xia, Tao

Xiao, Qinghan

Xie, Gaogang

Xie, Pu

Xie, Wen-fang

Xijun, Yan

Xilouris, Georgios

Xiong, Jijun

Xiong, Neal N.

Xiong, Shuping

Xiu, Shichao

Xu, Chunye

Xu, Huiping

Xu, Jian

Xu, Jiangtao

Xu, Jiaqiang

Xu, Jingkun

Xu, Lisheng

Xu, Longxia

Xu, Mingsen

Xu, Peiliang

Xu, Qiang

Xu, Qing-song

Xu, Wenyao

Xu, Xiang-min

Xu, Xiaodong

Xu, Xin

Xu, Yanliang

Xuan, Fu-zhen

Xuan, Xiangchun

Xue, Wei

Yacoot, Andrew

Yamashita, Masamichi

Yamazato, Takaya

Yamazoe, Noburu

Yan, Dong-Hang

Yan, Jize

Yan, Qingwen

Yan, Rongjin

Yang, Bai

Yang, Chia-min

Yang, Eui-Hyeok

Yang, Fan

Yang, Gongping

Yang, Hsiharng

Yang, Jie

Yang, Jin

Yang, Jinfeng

Yang, JL

Yang, Jun

Yang, Liang

Yang, Meng

Yang, Min

Yang, Ming

Yang, Nianjun

Yang, Ruigang

Yang, S. M.

Yang, S.H.

Yang, Shaowu

Yang, Shengyuan

Yang, Ting

Yang, Tsung-hsun

Yang, W.

Yang, Wei-hsiung

Yang, Ya-Ting

Yang, Yaoqing

Yang, Yongfeing

Yang, Yuh-shyong

Yang, Zhengpeng

Yang, Zhixin

Yao, Da-Jen

Yao, Hong

Yasui, Hiroyuki

Yavuz, Attila Altay

Yazdanparast, Razieh

Ye, Jianshan

Ye, Juan

Yen, Chih-Ta

Yen, Jia-yush

Yen, Ping-Lang

Yeo, Sang-Soo

Yi, Jingang

Yilmazer, Nuri

Yim, Jaegeol

Yin, Ming

Yin, Xue-Bo

Yin, Yilong

Yin, Yongmei

Yokokawa, Ryuji

Yoon, Sang Won

Yoon, Seog-young

Yoon, Seokho

Yoon, Seokhoon

Yoshie, Tomoyuki

Yoshikawa, Genki

Yoshioka, Hiroki

Yoshitomi, Yasunari

You, Changjun

Yousef, Hanna

Yu, Aimin

Yu, Chin-ping

Yu, Fei

Yu, Hongyu

Yu, Tang

Yu, Wen

Yu, Xia

Yu, Xiang

Yu, Xiong

Yuan, Bo

Yuan, Guangwei

Yuan, Junsong

Yuan, Ze-Chun

Yuasa, Masayoshi

Yuen, Peter

Yúfera, A.

Yuksel, Seniha Esen

Yun, Jaeseok

Yun, Kwang-Seok

Yun, Seok-Hyun

Zadok, Avi

Zagar, Bernhard

Zaharia, Titus

Zahn, Jeffrey D.

Zamorano, Jaime

Zampetti, E.

Zawadzki, Robert J.

Zazubovich, Valter

Zdunek, Artur

Zehnder, Pascal

Zemcik, Pavel

Zemčík, Robert

Zemp, Roger

Zeng, Caibin

Zeng, Tao

Zeze, Dagou

Zhan, Zili

Zhang, Amy H.

Zhang, Baochang

Zhang, Benniu

Zhang, Chun-yang

Zhang, Dan

Zhang, Dengfeng

Zhang, Dingguo

Zhang, Fei

Zhang, Gao-fei

Zhang, Guanglie

Zhang, Guangming

Zhang, Guohui

Zhang, Guoqiang

Zhang, Haibo

Zhang, Hao

Zhang, Jiantao

Zhang, Jinglan

Zhang, Lei

Zhang, Li

Zhang, Liangchi

Zhang, Pei

Zhang, Qianni

Zhang, Qijin

Zhang, Shu-Sheng

Zhang, Shujuan

Zhang, Song

Zhang, Tong

Zhang, Wei

Zhang, Weigang

zhang, wenming

Zhang, Wentao

Zhang, Xi

Zhang, Xiao-ping

Zhang, Xiaofei

Zhang, Xionghu

Zhang, Xuming

Zhang, Yafei

Zhang, Yan

Zhang, Yanping

Zhang, Yimin

Zhang, Ying

Zhang, Yong-Mei

Zhang, Yufeng

Zhang, Yulong

Zhang, Yuzhong

Zhang, Zhiqiang

Zhang, Zhongping

Zhao, Chaofang

Zhao, Haibo

Zhao, Hong

Zhao, Hui

Zhao, Jianlong

Zhao, Weian

Zhao, Xiangwei

Zhao, Xiaoming

Zhao, Ya-Pu

Zhao, Yanxiao

Zhao, Zuomin

Zheng, Rencheng

Zheng, Siyang

Zheng, Wei

Zheng, Yufeng

Zhi, Jinfang

Zhiyuan Liu, Zhiyuan

Zhong, Ziguo

Zhou, Bin

Zhou, Chuanhong

Zhou, Fengfeng

Zhou, Fuqiang

Zhou, Guanhua

Zhou, Huiyu

Zhou, Li-Hui

Zhou, Liang

Zhou, Sheng

Zhou, Susan

Zhou, Xiao Dong

Zhou, Xiaobing

Zhou, Xun (Sam)

Zhou, Yuanzhen

Zhou, Zhi

Zhou, Zhong

Zhu, Congyong

Zhu, Guoqiang

Zhu, Hu

Zhu, Hua

Zhu, Junjie

Zhu, Minghui

Zhu, Ning

Zhu, Qingzhi

Zhu, Shunpeng

Zhu, Weihong

Zhu, Xinqun

Zhu, Yuemin

Zhu, Zhigang

Zhu, Zhongkui

Zhuang, Guoqiang

Zhuo, Li

Zick, Kenneth M.

Zinnert, Julie

Zirak, Peyman

Zito, Domenico

Zogg, Hans

Zong, Changfu

Zorzi, Riccardo

Zotov, Sergei A.

Zou, Jie

Zou, Nan

Zsombor-Murray, Paul

Zumbado, Jennifer Rochlis

Zuo, Liang

Zvarec, Ondrej John

Zygmunt, Bogdan

Su, Hao

